# Rush Medical College

**Published:** 1870-03

**Authors:** 


### Editorial.

### *Rush Medical College — Spring and Summer Course.*

IN answer to numerous correspondents, we reply that this is not a regular or "graduating" course. It is conducted solely for purposes of instruction, for which this city furnishes most excellent opportunities.

